# Deep brain stimulation and motor cortex stimulation for central post-stroke pain: a systematic review and meta-analysis

**DOI:** 10.1093/pm/pnaf001

**Published:** 2025-01-11

**Authors:** Siddarth Kannan, Conor S Gillespie, Jeremy Hanemaaijer, John Eraifej, Andrew F Alalade, Alex Green

**Affiliations:** School of Medicine, University of Central Lancashire, Preston PR1 7BH, United Kingdom; Department of Clinical Neurosciences, University of Cambridge, Cambridge CB2 1PG, United Kingdom; Department of Neurosurgery, RadboudUMC, Nijmegen 6525GA, The Netherlands; Oxford Functional Neurosurgery Group, John Radcliffe Hospital, Oxford OX39DU, United Kingdom; Nuffield Department of Surgical Sciences, University of Oxford, Oxford OX1 2JD, United Kingdom; Oxford Functional Neurosurgery Group, John Radcliffe Hospital, Oxford OX39DU, United Kingdom; Nuffield Department of Surgical Sciences, University of Oxford, Oxford OX1 2JD, United Kingdom; School of Medicine, University of Central Lancashire, Preston PR1 7BH, United Kingdom; Department of Neurosurgery, Royal Preston Hospital, Preston PR2 9HT, United Kingdom; Oxford Functional Neurosurgery Group, John Radcliffe Hospital, Oxford OX39DU, United Kingdom; Nuffield Department of Surgical Sciences, University of Oxford, Oxford OX1 2JD, United Kingdom

**Keywords:** neuromodulation, post-stroke pain, deep-brain stimulation, motor cortex stimulation

## Abstract

**Introduction:**

Deep brain stimulation (DBS) and motor cortex stimulation (MCS) are invasive interventions in order to treat various neuropathic pain syndromes such as central post-stroke pain (CPSP). While each treatment has varying degree of success, comparative analysis has not yet been performed, and the success rates of these techniques using validated, objective pain scores have not been synthesized.

**Methods:**

A systematic review and meta-analysis was conducted in accordance with PRISMA guidelines. Three databases were searched, and articles published from January 2000 to October 2024 were included (last search date October 25, 2024). Meta-Analysis was performed using random effects models. We evaluated the performance of DBS or MCS by assessing studies that reported pain relief using visual analogue scale (VAS) or numerical rating scale (NRS) scores.

**Results:**

Of the 478 articles identified, 32 were included in the analysis (330 patients—139 DBS and 191 MCS). The improvement in mean VAS score for patients that underwent DBS post-surgery was 48.6% compared to a score of 53.1% for patients that had MCS. The pooled number of patients who improved after DBS was 0.62 (95% CI, 0.51–0.71, I2 = 16%). The pooled number of patients who improved after MCS was 0.64 (95% CI, 0.53–0.74, I2 = 40%).

**Conclusion:**

The use of neurosurgical interventions such as DBS and MCS are last-resort treatments for CPSP, with limited studies exploring and comparing these two techniques. While our study shows that MCS might be a slightly better treatment option, further research would need to be done to determine the appropriate surgical intervention in the treatment of CPSP.

## Introduction

Central post-stroke pain (CPSP) is one of the most challenging and distressing complications that stroke survivors face during recovery, affecting approximately 10%-39% of stroke patients.^1^^,^^2^ The onset of CPSP typically occurs within 1-3 months following a stroke, with the majority of cases manifesting symptoms by 6 months.^3^ This condition is characterized by chronic pain resulting from damage to the central nervous system, which severely impacts patients’ quality of life. The duration of CPSP can be chronic, lasting for months or even years after the initial stroke event. Studies suggest that CPSP affects approximately 8%-35% of stroke patients, with pain often persisting long after the stroke.^4^ The type of pain in CPSP is typically described as sharp, paroxysmal, and often localized to the hemiplegic side, though some patients report a more diffuse pain experience.^5^ Research indicates that stroke survivors with chronic pain often exhibit higher levels of depression and anxiety, which can exacerbate pain perception and complicate treatment.^2^

Currently, pharmacological treatments, such as anticonvulsants (eg, gabapentin), selective serotonin reuptake inhibitors (SSRIs), and antidepressants like amitriptyline, are commonly used to manage CPSP. However, these medications are often associated with significant side effects, especially at higher doses, and many patients are unable to tolerate these treatments.^6^ As a result, there is a growing need for alternative therapeutic options.

In recent years, invasive neuromodulation techniques have emerged as promising alternatives to manage CPSP, with deep brain stimulation (DBS) and motor cortex stimulation (MCS) at the forefront of innovative interventions.^7^ Both techniques involve the application of electrical impulses to specific brain areas, aiming to modulate neural circuitry and provide relief from chronic pain. However, the targeting areas for both interventions are different. In MCS, two electrode leads are placed over the motor and sensory cortices, while in DBS surgery, deeper located brain structures are targeted, such as the ventral posterolateral (VPL) and ventral posteromedial (VPM) nuclei of the thalamus, the periventricular and periaqueductal grey matter (PVG/PAG), or the rostral anterior cingulate cortex (ACC).^8^

While some studies have showcased this potential benefit, pooled comparative analysis has not yet been performed and the success rates of these techniques using validated, objective pain scores have not been synthesized. In this systematic review and meta-analysis, we aim to analyze the effect on pain relief offered by MCS and DBS on patients with CPSP using clearly defined outcomes.

## Methods

### Search strategy and selection criteria

We conducted this systematic review and meta-analysis according to the Preferred Reporting Items for Systematic Reviews and Meta-Analyses (PRISMA) Guidelines.^9^

We searched PubMed, Embase and Medline database of systematic reviews for full-text articles published in English (Search date October 25, 2024). Search terms used a combination of the terms “Central Post-Stroke Pain,” “Deep Brain Stimulation,” and “Motor Cortex Stimulation,” and their associated synonyms. The full search strategy for all databases can be found in Supplementary Tables S1–S3. The Population, Intervention, Comparator, Outcome, Study Design (PICOS) criteria was used (Supplementary Table S4). Furthermore, excluded reviews and the reference list of retrieved articles were cross-referenced for enriching and completing the included database. We included studies of adults (≥18 years) that specifically mentioned the use of either DBS or MCS for the treatment of CPSP. We excluded studies that reported exclusively pediatric populations, and studies that examined other forms of neuropathic pain such as trigeminal neuralgia, diabetic and peripheral neuropathy. We excluded studies that were conference abstracts or if the primary language was not English.

Two reviewers (S.K. and C.S.G.) independently screened titles, abstracts and full texts to include articles. If reviewers failed to reach consensus, a third author was sought for clarification.

### Data extraction

Data extraction was completed by two authors independently (S.K. and C.S.G.). The following data were extracted from included studies: Year published, journal, type of study (Randomized Control Trial [RCT] or observational study), single/multi center, number of patients with CPSP, and number of these patients that underwent either MCS or DBS, number of patients that saw an improvement in pain, mean postoperative visual analogue scale (VAS) or numerical rating scale (NRS) scores and mean follow up time. The VAS or NRS score was used as primary outcome metric in this study, offering a validated and standardized measure of pain intensity; the scale in each study was measured in 0-10. Any study that did not report individual patient NRS/VAS improvement scores were excluded.

### Risk of bias assessment

Risk of bias assessment was completed by two reviewers independently (S.K. and C.S.G.). Retrospective studies were classified according to the Newcastle-Ottowa Scale (NOS).^10^^,^^11^ NOS is a tool used to assess the quality of non-randomized studies, particularly cohort and case–control studies. Evaluation is based on three broad criteria: selection, comparability, and outcome (for cohort studies) or Exposure (for case–control studies). Each of these criteria includes several sub-criteria, with points awarded to studies based on how well they meet each criterion.

### Statistical analysis

Baseline characteristics were presented as descriptive frequencies. For meta-analysis, we used random effects models of variables and endpoints, with pooled proportions used for the reduction of pain scores using VAS as a continuous outcome measure. We evaluated the performance of DBS or MCS by assessing studies that reported pain relief using VAS or NRS scores. Pain improvement was defined as a reduction of ≥30% on the VAS or NRS score. This is considered clinically significant, as previous studies have indicated that reductions in pain scores of around 30%-40% are needed to reflect clinically useful improvements.^12^^,^^13^ The 30% cut-off for defining improvement was consistently applied across all studies included in this analysis.

The total number of patients in each study, along with the number of patients who experienced pain improvement of ≥30%, was extracted. These data were then pooled by calculating proportions representing the percentage of patients experiencing clinically significant pain relief. The pooled proportions were aggregated across studies using random effects models to provide an overall estimate. A 95% confidence interval (CI) was calculated to account for study variability and quantify the uncertainty around the estimated proportions. This approach allowed us to evaluate the clinical effectiveness of the interventions across different studies, providing a measure of precision for the pooled estimates and reflecting the range within which the true proportion is expected to lie 95% of the time.

We carried out an additional sensitivity analysis by selectively removing studies at high risk of bias, then re-running the meta-analysis.

Data analysis of descriptive statistics was performed using the software Statistical Package for the Social Sciences (version 27; IBM; Armonk; NY). R statistics (Rstudio Version 4.0.1) was used to perform a meta-analysis and create figures, forest, and funnel plots (ggplot2 and meta-packages).

For each random effects model, we tested heterogeneity using the maximum restricted likelihood estimator. Prevalence was calculated using pooled proportions methods using the inverse variance method. The *I*^2^ statistic was used to quantify the percentage of total variation across studies that is due to heterogeneity rather than chance.^14^  *I*^2^ values were interpreted as follows: 0%-25%: Low heterogeneity, 26%-50%: Moderate heterogeneity, >50%: Significant heterogeneity.^15^ Heterogeneity was considered significant when *I*^2^ > 50% and the *P* value < 0.1. In cases of significant heterogeneity, further investigation was conducted to explore the sources of variability among studies. Publication bias was assessed using Egger’s test and by inspection of funnel plots.

### Sensitivity analysis

Further analysis was performed by including studies with a minimum of only five patients in order to assess if there was any impact on the overall results.

## Results

### Study details

**Figure 1.**
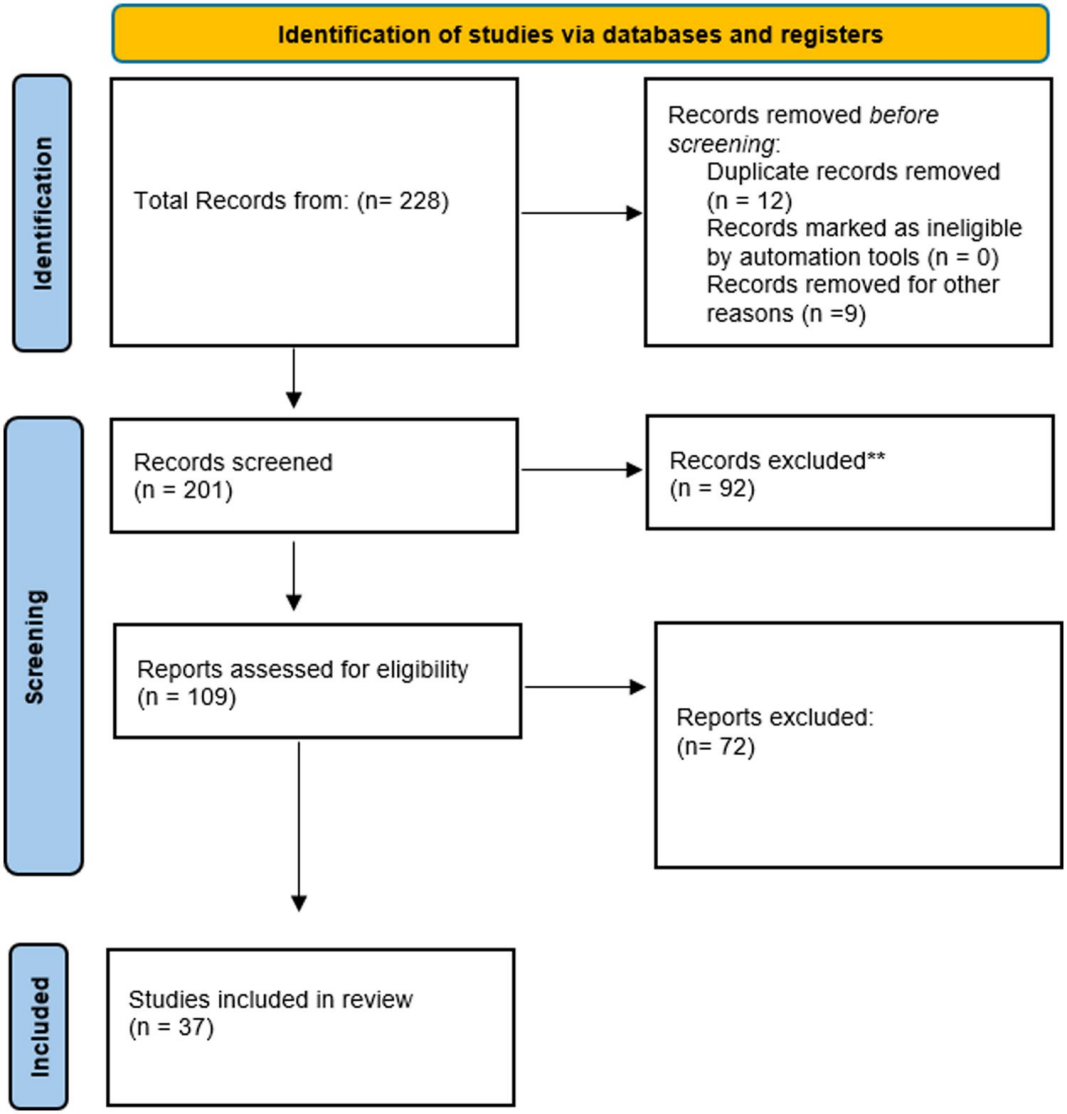
PRISMA Flow diagram, of study selection for inclusion in this review and meta-analysis.

After removal of duplicates, 97 studies were identified. After full-text assessment, 37 full-text studies were assessed for inclusion and were finally included, shown in Figure 1 (Supplementary Table S5). In total, 32 studies were included in the meta-analysis, after removing five case reports.

### Baseline characteristics

The baseline characteristics of included studies are summarized in Table 1 with a total number of 330 patients. The most common country of published studies was the United Kingdom (26.3%%, *n* = 10). Among the 32 studies included, 16 studies explored the effects of DBS and 16 studies the outcome of MCS. Mean follow up for DBS studies was 18.7 months. For MCS studies, seven studies reported a follow-up time, with a mean of 22.3 months.

**Table 1.** Baseline characteristics.

Characteristic	*N* (%)
Country of origin	
UK	10 (26.3%)
France	5 (13.1%)
USA	4 (10.5%)
Germany	3 (7.9%)
Japan	3 (7.9%)
Italy	2 (5.3%)
China	2 (5.3%)
Poland	2 (5.3%)
Russia	1 (2.6%)
Belgium	1 (2.6%)
Netherlands	1 (2.6%)
Canada	1 (2.6%)
South Korea	1 (2.6%)
Switzerland	1 (2.6%)
Study design	
Retrospective	33 (100%)
Total number of patients	330
Total number of improved patients	210
DBS	
Number of patients	139
Number of patients showed improvement in pain relief	87 (62.5%)
Mean VAS score improvement post-surgery	48.6% (±13.42%)
Mean follow up time in months (Standard deviation)	18.67 (±13.52)
MCS	
Patients	191
Number of patients showed improvement in pain relief	123 (64.3%)
Mean VAS score improvement post-surgery	53.17% (±9.27%)
Mean follow-up time in months (Standard deviation)	22.3 (±10.1)

### Effect of MCS and DBS on VAS pain relief

**Figure 2.**
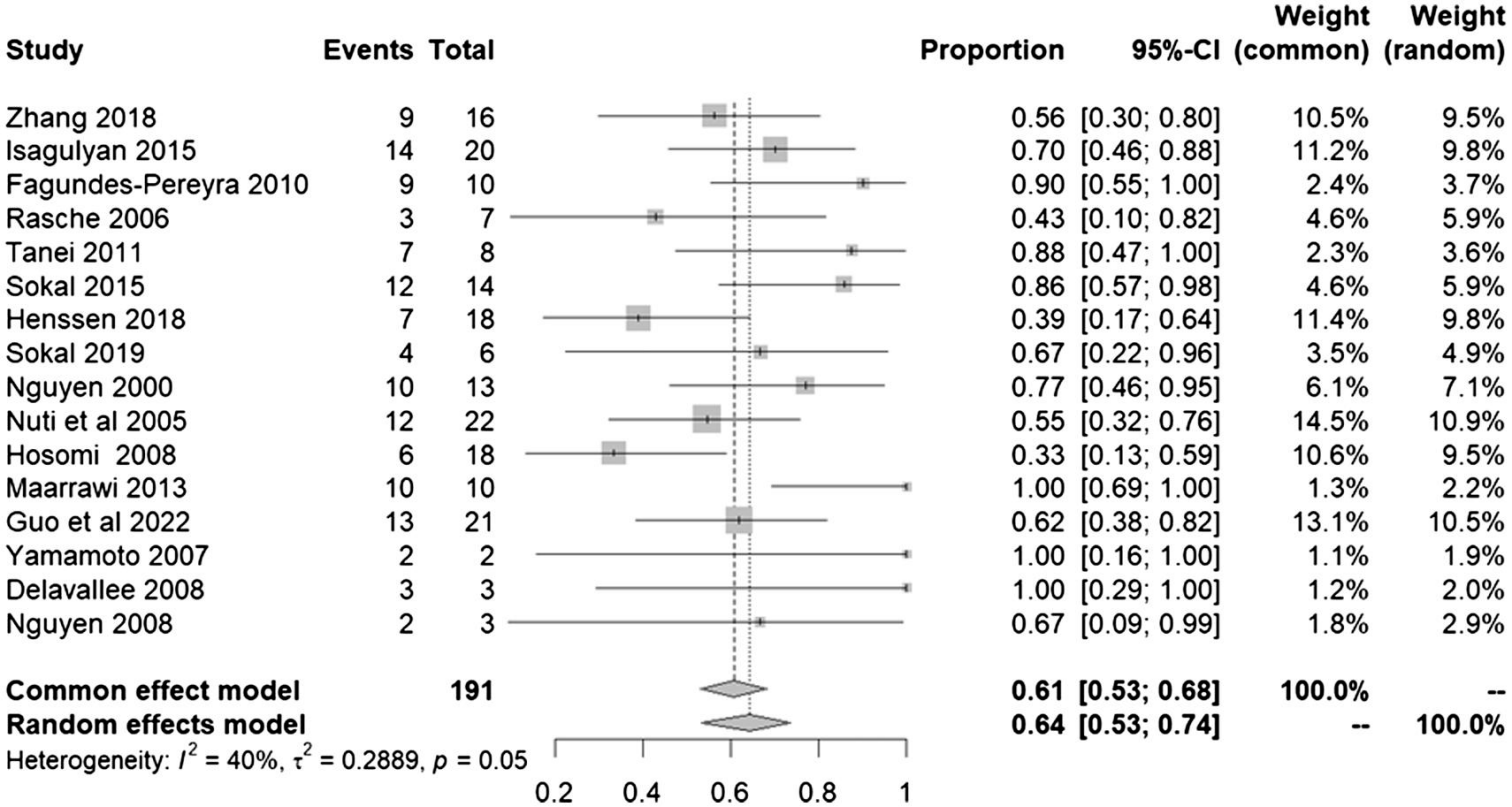
Forest plot showcasing VAS improvement after MCS using and random effects models.

The analysis of Sixteen MCS studies involving a total of 195 patients,^1^^,^^6^^,^^16–29^ revealed that 0.62 (95% CI: [0.53–0.74], *I*^2^ = 40%) experienced pain improvement after undergoing MCS (Figure 2).

**Figure 3.**
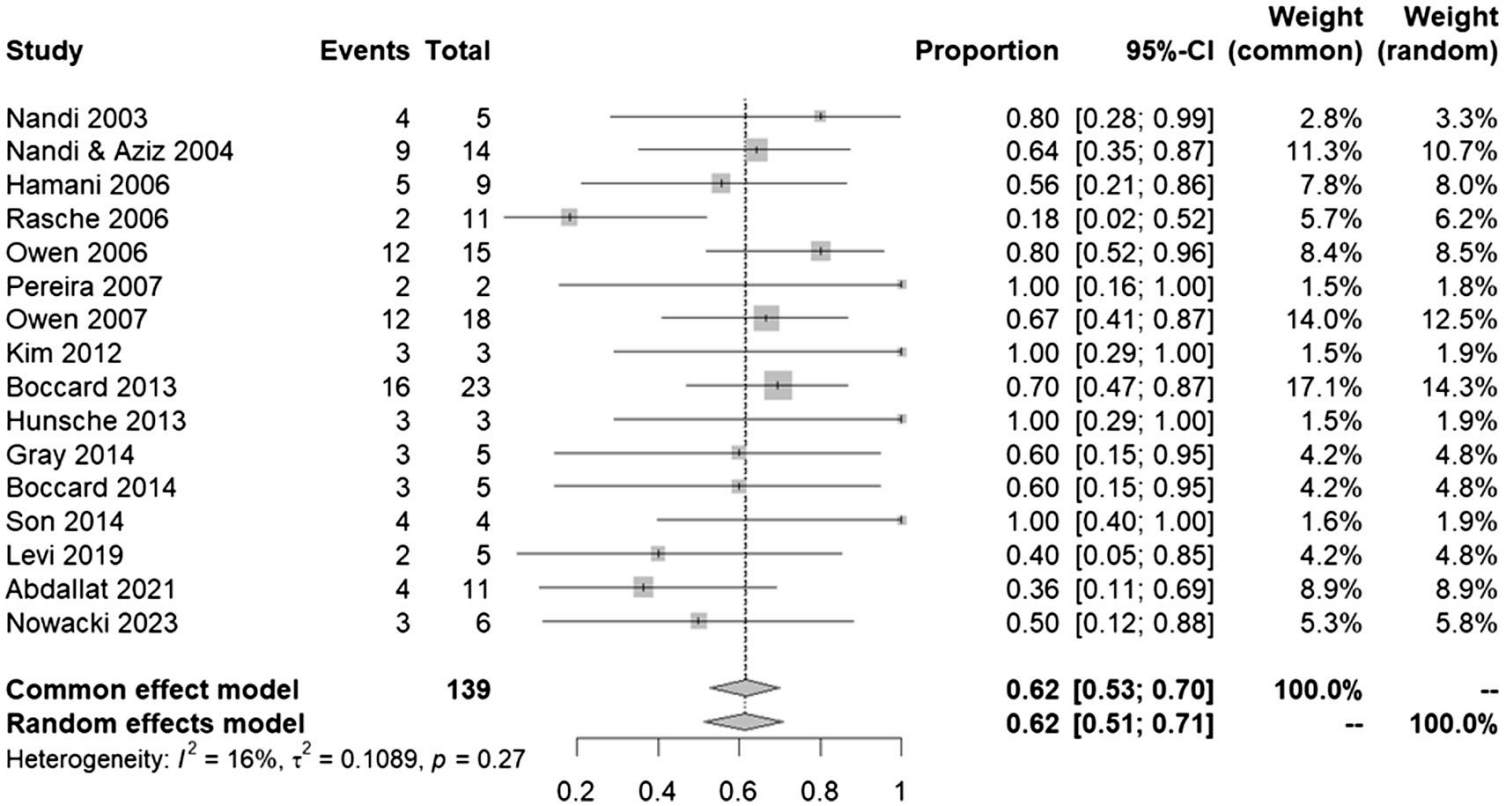
Forest plot showcasing VAS improvement after DBS using random effects models.

Among the sixteen DBS studies included that could be pooled for the meta-analysis,^30–45^ the number of patients who have shown an improvement out of each cohort is presented in Figure 3. The pooled proportion of patients whose pain scores improved after DBS was 0.62 (95% CI, 0.51-0.71, *I*^2^=16%).

**Figure 4.**
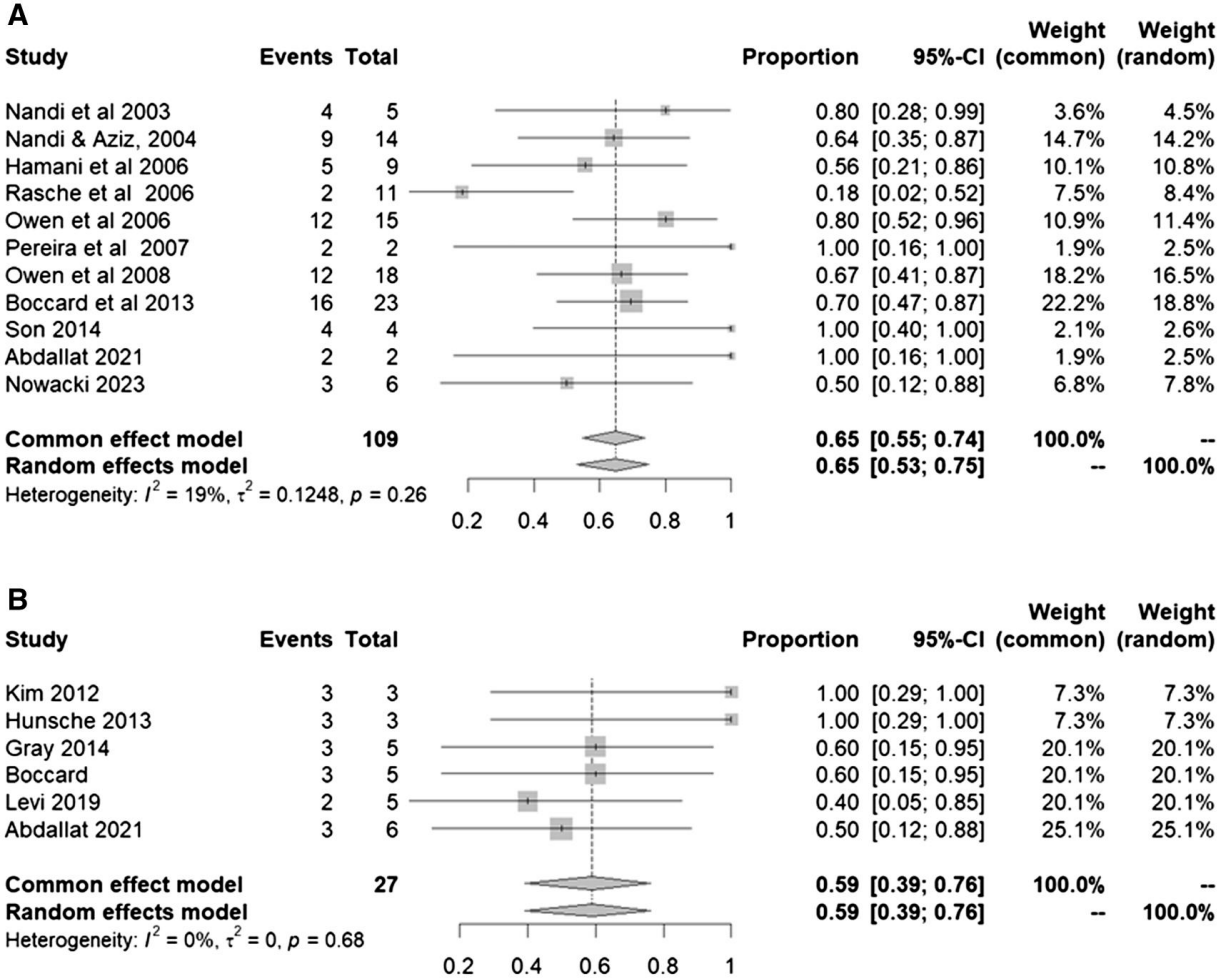
(A) Forest plot showcasing VAS improvement in studies targeting the PVG/VPL using random effects models. (B) Forest plot showcasing VAS improvement in various other targets using random effects models.

Further analysis was conducted based on the stimulation target. Majority of the studies (10/16) targeted the PVG and VPL thalamic nucleus in 108 patients (Figure 4A). Across these studies, two electrodes are placed, one each in the PVG and VPL unilaterally. The placement of the electrodes is contralateral to the pain affected regions.

The remaining studies targeted the anterior cingulate cortex (ACC) in 11 patients, and the posterior limb of the internal capsule (PLIC) in 10 patients. Six patients in the centromedian–parafascicular nucleus were also included.^30^ No significant conclusion regarding the optimal site for pain reduction could be made due to the fewer number of studies and smaller sample size (Figure 4B).

### Sensitivity analysis

In DBS, 12 studies met the criteria for sensitivity analysis compared to thirteen MCS studies. Removing four studies^35^^,^^36^^,^^43^^,^^45^ studies reduced the improvement in pain relief post-DBS implantation to 0.59 (95% C1: 0.48–0.69, *P* = 0.24) from 0.624 (95% CI: 0.51–0.71, *P* = 0.27). This was similar for the MCS cohort where the removal of three studies,^1^^,^^22^^,^^28^ reduced the improvement in pain relief to 0.63 (95% C1: 0.52–0.72, *P* < 0.05) from 0.64 (95% CI: 0.53–0.74, *P* = 0.05) (Supplementary Figures S1 and S2).

### Egger’s test

**Figure 5.**
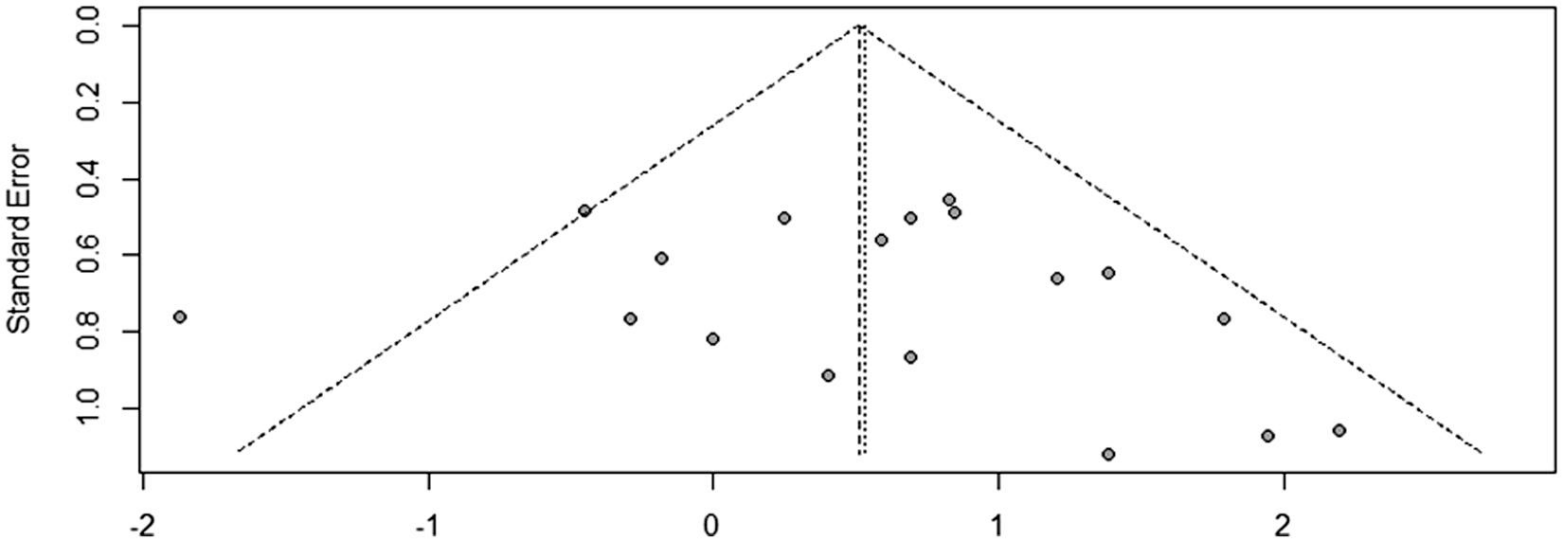
Funnel plot assessing publication bias.

The potential for publication bias in this meta-analysis was assessed using a funnel plot and Egger’s regression test. The funnel plot demonstrated a symmetrical distribution of study effect sizes around the central line, indicating no obvious visual signs of asymmetry (Figure 5). To statistically evaluate this, Egger’s test was performed, yielding a non-significant result (*t* = 0.81, df = 17, *P* = 0.4297). This *P* value, which is well above the conventional threshold of 0.05, suggests no statistically significant asymmetry in the funnel plot. Consequently, these findings provide no strong evidence of publication bias within the studies included in this analysis. Thus, it can be reasonably concluded that the results of this meta-analysis are unlikely to be influenced by publication bias. Four studies were excluded from the Egger’s test due to missing standard error.

### Risk of bias

**Figure 6.**
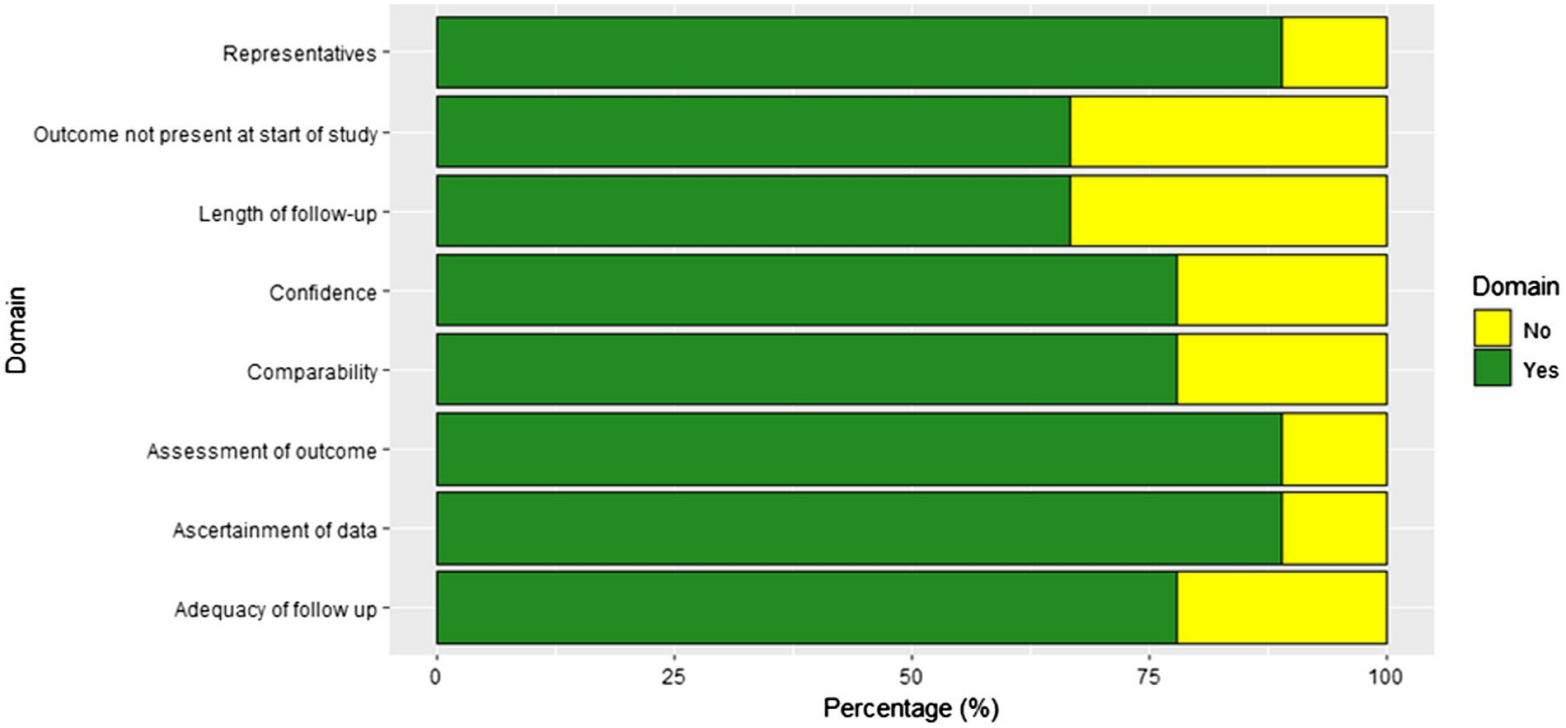
Risk of bias using the Newcastle–Ottowa scale.

The Risk of bias for retrospective cohort studies, using the Newcastle-Ottawa Scale. The mean score for all studies was 7.5 (out of a total maximum score of 9), and 5 studies were classified as high risk of bias (Figure 6).

## Discussion

This systematic review and meta-analysis is the first to pool the effect of MCS and DBS in patients with CPSP. By assessing the 32 studies, we found that MCS has an improvement in pain in 64.3% (123/191) of the patients and 62.5% (87/139) in patients receiving DBS. Patients that underwent MCS had a mean VAS improvement of 53.1% compared to 48.6% in patients that underwent DBS.

Several theories about CPSP have been proposed. It is believed that CPSP could be caused by an imbalance between the paleospinothalamic (affective-emotional) and neospinothalamic (sensory-discriminative) pathways. Evidence indicates that impaired spinothalamic tract function is related to the pathogenesis of CPSP.^46^ It has been hypothesized that MCS decreases thalamic hyperactivity by inducing corticothalamic connections.^47^^,^^48^ However, the role of N-methyl-D-aspartate (NMDA) receptors and GABAergic interneurons has also been proposed as an interesting aspect of the potential mechanism in MCS.^3^^,^^49^^,^^50^ Repetitive Transcranial Magnetic Stimulation (rTMS) of the motor cortex has shown restoration of intracortical inhibition in neuropathic pain patients, where the degree of pain relief correlates with the amount of restoration of inhibition.^51^ In addition, activation of the endogenous opioid descending system and alterations in the limbic system are described as potential mechanisms in MCS.^52^^,^^53^

As in MCS, the precise pain-relieving effect of DBS remains incompletely elucidated. The main difference between DBS and MCS is that the effect ensured through stimulation in DBS varies depending on the targeted brain area. Pain improvement in VPL/VPM thalamus DBS could be caused by the alteration of the balance of excitatory and inhibitory neurotransmitters within the pain pathways,^54^^,^^55^ which partly aligns with the potential underlying mechanisms of MCS. Stimulating the PVG and PAG will result in releasing endogenous opioid peptides with a decrease in activity of nociceptive signal-transmitting neurons.^54^^,^^55^ An alteration of the emotional and cognitive aspects of chronic pain is suggested when stimulating brain targets of the limbic system, such as the ACC.^54^^,^^55^ The lack of insight in the underlying mechanisms of DBS and MCS, as well as the pathophysiology of CPSP, illustrates the complexity of this pain syndrome and the need for a multidimensional approach in pain modulation therapies.

While the literature is limited on the direct comparison of DBS vs MCS on CPSP, several studies have scrutinized its efficacy individually. A study by Owen et al.,^41^ examined the effect of DBS on 47 patients with CPSP and found a mean improvement in VAS score of 59%. Studies have found varying results with mean VAS pain relief of between 38.1% and 68.4%.^33^^,^^36^^,^^56^ However, determinant factors should be taken into consideration, such as heterogeneity in terms of targeted areas: PVG and VPL were the most common sites with two studies targeting the ACC and one study each targeting the PLIC. Studies looking at the effect of MCS are limited in comparison with DBS. A study by Zhang et al.^29^ looked at the effect of MCS on 16 patients with CPSP and found a mean improvement in VAS score of 42.3%. Similar to patients who underwent DBS surgery, MCS has been shown to improve VAS scores by 40%-63.8%.^57^^,^^27^

A study by Nandi et al.,^58^ analyzed the use of MCS and VPL/PVG DBS on patients with CPSP. This study concluded that while MCS offers better pain relief, this varies between patients and is inconsistent in the long-term outcome.^58^ Another study by Katayama et al.,^59^ found that a greater proportion of MCS patients experienced pain relief compared to ventral caudalis (VC) DBS patient. Both studies indicated that DBS is a simpler procedure and generally better tolerated in patients.

Similar results were also found in studies comparing MCS vs DBS in other forms of neuropathic pain. Son et al.,^45^ directly analyzed MCS and DBS in the same eight patients with chronic intractable neuropathic pain. MCS was successful in reducing pain in 6/8 compared to 2/8 in DBS.^45^

While our results vary slightly with the current literature on mean VAS score, this could be attributed to various factors such as a larger total population of 330 pooled into the analysis, with other studies varying between 6 and 47 patients. As previously mentioned, the site of DBS insertion is key and could have played an important role in the heterogeneity of the population.

### Clinical and research implications

Our results have several implications for research and clinical practise. The slightly better success of MCS, solely based on pain scores, could aid clinicians in determining its appropriate use. However, multiple factors should be taken into account before utilizing MCS over DBS as a last-resort treatment for CPSP, including the risk profile, side effects, and patients’ medical history.

The practical implications, such as treatment cost, could play a crucial role in determining the most suitable intervention, given the similar success-rates. While there are currently no studies directly comparing the cost-effectiveness of DBS and MCS for neuropathic pain, a cost analysis study by Zaghi et al.^60^ indicated that MCS incurs significant initial expenses, estimated at $42 000.00. After 1-year of follow-up, with monthly visits to assess the parameters and configurations, the total cost of treatment is estimated to be around $45 600.00.^60^ In comparison, an analysis by Bishay et al.^61^ of DBS costs across various disorders, neuropathic pain not included, estimated an inflation- and currency-adjusted mean cost of $40 942.85 ± $17 987.43 for total DBS surgery. The initial cost of DBS treatment increases to $47 632.22 ± $23 067.08 after 1-year of follow-up.^61^ Regardless, this is not a direct in-depth cost-effectiveness analysis between MCS and DBS for CPSP. It provides an impression of the estimated total costs for both interventions, including the first year of intensive follow-up.

Follow-up care is essential for optimizing treatment outcomes in both interventions. Although the nature and intensity of follow-up can vary significantly within patients. The “trial and error” approach to programming different configurations and parameters in patients treated with MCS or DBS is time consuming and patient specific. Therefore, novel research on investigating connectivity-based predictive models could potentially address these challenges. A personalized approach that optimizes configuration and parameters to target specific brain networks may improve the future application of MCS or DBS in the treatment of CPSP.

Generally, DBS is considered more invasive than MCS. This procedure involves the implantation of electrodes into deeper brain regions, while MCS requires electrode placement on the surface of the dura mater. Nevertheless, a craniotomy has been carried out over the Rolandic region for appropriate placement in MCS, whereas a burr hole approach is used in DBS surgery.

Although MCS and DBS are distinct procedures, adverse events appear to be rare and manageable. Potential adverse effects following DBS and MCS surgery include seizures, hematomas, infections, headaches and hardware malfunction. In addition, some complications are procedure specific, such as epidural fibrosis, electrode migration, and effusion formation are associated with MCS surgery.^62^ In DBS, the side effects are more location dependent, such as paraesthesias, muscle spasms, and phosphenes. Side effects could also occur at accustomed therapeutic voltages, the electrode leads therefore should be repositioned into a slightly altered location.^63^ The aforementioned findings highlight that while DBS and MCS are effective therapies for chronic neuropathic pain, careful and accurate post-surgical management is not only required for optimizing result, but also cost-effectiveness and minimizing the risk of adverse events.

In contrast to the invasive nature of DBS and MCS, non-invasive brain stimulation (NIBS) techniques have been investigated to adjust the excitability of specific functional brain regions.^64^^,^^65^ The most prevalent utilized NIBS techniques in clinical setting are transcranial magnetic stimulation (TMS) and transcranial direct current stimulation (tDCS).

Research indicates that rTMS can effectively reduce neuropathic pain. A study demonstrated that rTMS targeting the motor cortex resulted in significant pain relief for patients suffering from refractory neuropathic pain.^66^ The mechanism described by which rTMS alleviates pain is thought to involve modulation of cortical excitability and restoration of normal brain function in pain processing pathways.^67^ A clinical trial found that anodal tDCS applied to the motor cortex significantly ameliorated chronic pain and reduced intracortical inhibition, suggesting a potential mechanism for its analgesic effects.^68^ While there is no study directly comparing the effect of NIBS on CPSP, a recent meta-analysis on sensory function recovery in stroke patients showed that both tDCS and rTMS significantly outperformed control conditions. NIBS was beneficial in the acute and subacute phases of stroke, while a moderate effect was observed in chronic stroke patients.^69^

Although rTMS currently has been described as a predictive factor for MCS, the application of NIBS treatments could potentially being integrated in the treatment of CPSP after warrant future research. Moreover, the integration of individualized treatment protocols is likely to play a crucial role in the future of non-invasive neuromodulation.^70^ Current research suggests that the same stimulation parameters may not yield uniform effects across different individuals due to variations in brain anatomy and neurophysiology.^71^ Personalized approaches, such as adjusting stimulation intensity and targeting specific brain regions based on individual patient profiles, could enhance treatment outcomes and minimize side effects.^72^

Though we analyze the efficacy of DBS and MCS on patient outcome there are certain aspects that need to be addressed. The success of MCS could be due to fewer studies present on this topic and the impact of information bias would need to be considered. Various factors impacting pain relief such as severity and location of stroke, age, and social habits could have an impact on the overall outcome.^73^^,^^74^ Studies have also shown that coping strategies such as social support can influence the effect of pain relief.^75^^,^^76^ A cross-sectional study found that access to a trusted healthcare professional, living with pain for ≥10 years and polypharmacy had a significant effect on the amount of pain relief.^77^

### Limitations

This study has several limitations. Firstly, all studies included were retrospective, precluding pooled analysis of prospective studies. In addition, the region of the brain where DBS was performed is heterogeneous and could have impacted the effect of pain relief. In MCS, the variety of surgical approaches over time and the exact location of the electrode leads could influence the level of pain relief due to the lack of a standardized protocol. In this meta-analysis, only pain scores have been considered, whereas in pain research, Quality of Life (QoL) and medication use is at least as important. We also excluded full-text papers not available in English, restricting paper eligibility.

## Conclusion

The use of neurosurgical interventions, such as DBS and MCS, are a propitious field for the treatment of CPSP, with limited studies exploring and comparing these two techniques.

While our study suggests a modest improvement in pain scores with MCS over DBS in patients suffering from CPSP, these findings should be viewed as preliminary. Pain scores alone is insufficient to establish MCS as a definitively superior or non-inferior treatment option compared to DBS. Further factors such as cost-effectiveness in pain treatment, long-term efficacy and multi-dimensional functional outcomes would need to be assessed in order to determine the appropriate surgical neuromodulation for CPSP.

## Supplementary Material

pnaf001_Supplementary_Data

## Data Availability

The original contributions presented in the study are included in the article, further inquiries can be directed to the corresponding author.
