# Supplementary material for: Deep brain stimulation and motor cortex stimulation for central post-stroke pain: a systematic review and meta-analysis
Source: Pain Med. 2025 Jan 11;26(5):269–78. doi: 10.1093/pm/pnaf001 (PMC12046226; doi:10.1093/pm/pnaf001)
Supplement: pnaf001_Supplementary_Data [file pnaf001_supplementary_data.docx]

**Supplementary Table 1.OVID Medline search strategy Search performed 20^th^ October 2024).**

| **Search** | **Query** | **Results** |
| --- | --- | --- |
| 1 | exp Deep Brain Stimulation/ | 10922 |
| 2 | Motor Cortex Stimulation.mp. | 702 |
| 3 | 1 or 2 | 11289 |
| 4 | (Post stroke pain OR Post-stroke Pain OR Central Post-stroke Pain OR Central Post stroke Pain) | 358 |
| 5 | 3 AND 4 | 59 |
| 6 | Remove duplicates from 5 | 49 |
| 7 | 6 not (animals not humans) | 49 |
| 8 | Limit 7 to English | 49 |

**Supplementary Table 2. OVID Embase search strategy Search performed 20^th^ October 2024).**

| **Search** | **Query** | **Results** |
| --- | --- | --- |
| 1 | exp Deep Brain Stimulation/ | 12287 |
| 2 | Motor Cortex Stimulation.mp. | 902 |
| 3 | 1 or 2 | 12559 |
| 4 | (Post stroke pain OR Post-stroke Pain OR Central Post-stroke Pain OR Central Post stroke Pain) | 572 |
| 5 | 3 AND 4 | 114 |
| 6 | Remove duplicates from 5 | 101 |
| 7 | 6 not (animals not humans) | 97 |
| 8 | Limit 7 to English | 95 |

**Supplementary Table 3. Pubmed search strategy (Search performed 20^th^ October 2024).**

| **Search** | **Query** | **Results** |
| --- | --- | --- |
| 1 | (Deep brain stimulation OR DBS) OR (Motor cortex stimulation OR MCS) AND (Post stroke pain OR Post-stroke Pain OR Central Post-stroke Pain OR Central Post stroke Pain) | 101 |
| 2 | Limit to English | 84 |
| 3 | Limit to Animals | 79 |

**Supplementary Table 4. PICOS inclusion criteria**

| **Review Question** | In patients with Post stroke pain, does Deep brain stimulation or motor cortex stimulation offer better pain relief? | |
| --- | --- | --- |
| **Population** | Adults ≥ 18 years diagnosed with central post stroke pain. | |
| **Intervention** | Deep brain stimulation and Motor cortex stimulation | |
| **Comparator** | None | |
| **Outcomes** | **Primary** | **Secondary** |
|  | VAS improvement offered by DBS | Prevalence of post stroke pain |
|  | VAS improvement difference based on different target site by DBS | Number of studies offering pain relief data (via VAS) in patients treated via MCS/DBS |
|  | VAS improvement offered by MCS |  |
| **Setting** | Studies taking place in any neurosurgical department | |
| **Study design** | Phase 3 trials, prospective case series and cohort studies with >1 adult patient | |

**Supplementary Table 5 .DBS & MCS Study characteristics**

| Author | Study design | Country | Mean Age | Total Number of patients | Gender | Improved patients | Mean VAS pain relief | Stimulation target |
| --- | --- | --- | --- | --- | --- | --- | --- | --- |
| Nandi et al., 2003 | Case series | UK | 58.4 | 5 | M:3  F:2 | 4 | 37.25% | PVG and the ventroposterolateral thalamic nucleus |
| Nandi & Aziz, 2004 | Case series | UK | N/A | 14 | N/A | 9 | 68.40% | PVG and the ventroposterolateral thalamic nucleus |
| Hamani et al., 2006 | Retrospective cohort | Canada | 64.4 | 9 | M:3  F:6 | 5 | 62% | ventrocaudalis thalamic nucleus and the PAG/PVC |
| Rasche et al., 2006 | Case series | USA | N/A | 11 | N/A | 2 | 50% | PVG and the ventroposterolateral thalamic nucleus |
| Owen et al., 2006 | Case series | UK | 58.6 | 15 | M:12  F:3 | 12 | 36.90% | PVG and the ventroposterolateral thalamic nucleus |
| Pereira et al., 2007 | Case series | UK | 62 | 2 | F:2 | 2 | 38.50% | PVG and the ventroposterolateral thalamic nucleus |
| Owen et al., 2007 | Cohort | UK | 50 | 18 | N/A | 12 | 49% | PVG and the ventroposterolateral thalamic nucleus |
| Franzini et al., 2008 | Case report | Italy | 58 | 1 | M:1 | 1 | 40% | posterior limb of the internal capsule (IC) |
| Alves & Asfora, 2011 | Case report | USA | 53 | 1 | M:1 | 1 | 60% | left centromedian thalamic nucle |
| Kim et al., 2012 | Case series | Korea | 51.7 | 3 | M:1  F:2 | 3 | 68.40% | unilateral ventralis caudalis |
| Hunsche et al., 2013 | Case series | Germany | 61.0 | 3 | M:2  F:1 | 3 | 30% | posterior limb of the internal capsule (PLIC) |
| Boccard et al., 2013 | Cohort | UK | 52.4 | 23 | M:16  F:7 | 16 | 38.10% | PAG/VPL |
| Gray et al., 2014 | Case series | UK | 50.4 | 5 | M:4  F:1 | 3 | 57.70% | PVG/PAG |
| Boccard et al., 2014 | Case series | UK | 51 | 5 | M:3  F:2 | 3 | 32.30% | anterior cingulate cortex (ACC) |
| Son et al., 2014 | Case series | USA | 57.5 | 4 | M:2  F:2 | 4 | 42.40% | PVG/PAG |
| Rezaei Haddad et al., 2015 | Case report | UK | 52 | 1 | M:1 | 1 | 40% | ventrocaudalis parvocellularis internis |
| Holland et al., 2018 | Case report | US | 48 | 1 | F:1 | 1 | 62% | PVG/PAG |
| Levi et al 2019 | Cohort | Italy | 56.2 | 5 | M:4  F:1 | 2 | NRS improvement:  37.9% | anterior cingulate cortex (ACC) |
| Abdallat et al 2021 | Cohort | Germany | 54.5 | 11 | M:7  F:4 | 4 | 46.3% | N/A |
| Nowacki et al 2023 | Cohort | Switzerland | 51.3 | 6 | M:3  F:3 | 3 | NRS improvement: 50% | PVG/PAG |

| Author | Study type | Country | Mean Age | Gender | Total Number of patients | Improved patients | Mean VAS pain relief |
| --- | --- | --- | --- | --- | --- | --- | --- |
| Nussel et al., 2020 | Case report | Germany | 62 | M: 1 | 1 | 1 | 40% |
| Zhang et al., 2018 | Cohort | China | 59.9 | M:8  F:8 | 16 | 9 | 42.30% |
| Isagulyan et al., 2015 | Cohort | Russia | 61 | M:13  F:7 | 20 | 14 | 36% |
| Fagundes-Pereyra et al.,2010 | Cohort | France | 61.3 | M:7  F:3 | 10 | 10 | 58.20% |
| Rasche et al., 2006 | Cohort | UK | 65.1 | M:4  F:3 | 7 | 3 | 43% |
| Tanei et al.,2011 | Cohort | Japan | 59.4 | M:5  F:3 | 8 | 7 | 63.80% |
| Yamamoto et al., 2007 | Case report | Japan | 66.5 | M:1  F:1 | 2 | 2 | 52% |
| Sokal et al.,2015 | Cohort | Poland | 58 | M: 8  F: 6 | 14 | 12 | 46% |
| Henssen et al., 2018 | Cross-sectional | Netherlands | 59 | M: 10  F: 8 | 18 | 7 | 53.10% |
| Sokal et al., 2019 | Cohort | Poland | 61.2 | M: 4  F: 2 | 6 | 4 | 48% |
| Nguyen et al., 2000 | Cross-sectional | France | 54 | N/A | 13 | 10 | 52.30% |
| Nuti et al 2005 | Cohort | France | 57.3 | M: 9  F: 13 | 22 | 12 | 63.7% |
| Delavallée et al 2008 | Case series | Belgium | 64 | M:1  F:2 | 3 | 3 | 72% |
| Hosomi et al 2008 | Cohort | Japan | 57.9 | M:14  F:4 | 18 | 6 | 35.8% |
| Nguyen et al 2008 | Case series | France | 59.7 | M:1  F:2 | 3 | 2 | 44.7% |
| Maarrawi et al 2013 | Cohort | France | 49.9 | M:6  F:4 | 10 | 10 | 51% |
| Guo et al 2022 | Cohort | China | 54.6 | M: 7  F:14 | 21 | 13 | 35.9% |


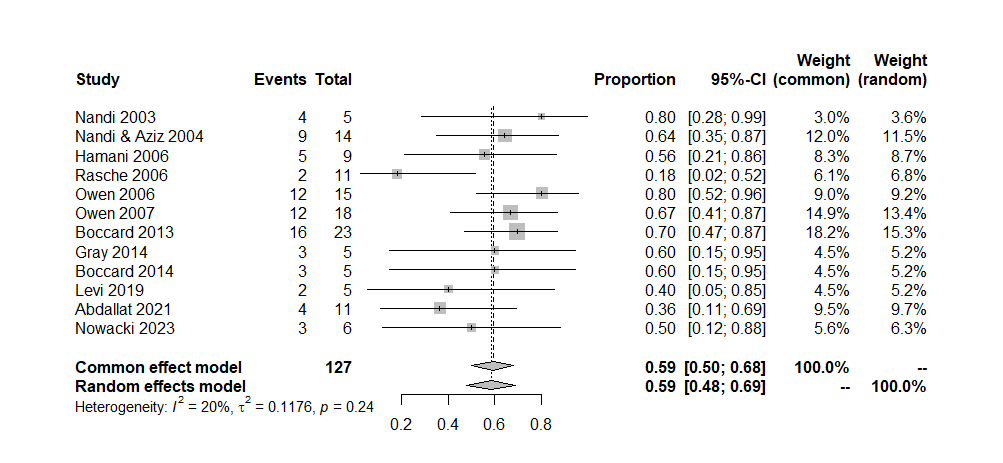


**Supplementary Figure 1: Sensitivity analysis showcasing VAS improvement after DBS using random effects models.**


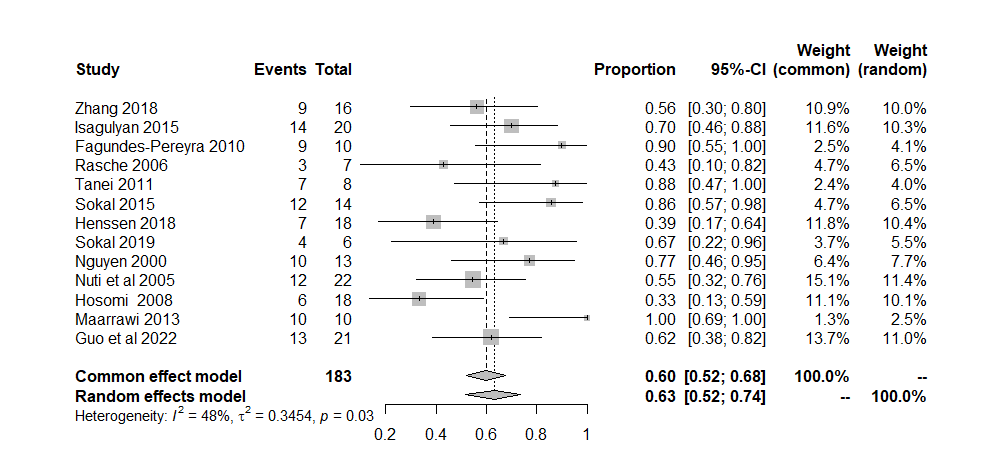


**Supplementary Figure 2: Sensitivity analysis showcasing VAS improvement after MCS using random effects models**
